# Supplementary material for: The landscape of Arabidopsis tRNA aminoacylation
Source: Plant J. 2024 Nov 18;120(6):2784–802. doi: 10.1111/tpj.17146 (PMC11658184; doi:10.1111/tpj.17146)
Supplement: Supplementary file 1 — Figure S1. Pairwise matrix showing correlation in read abundance measurements for each library. Figure S2. Variation among Arabidopsis tRNA isodecoder families and genomic compartments in CCA tail integrity in response to periodate treatment. Figure S3. Variation among human cell line (HEK293T) tRNA isoacceptor families and genomic compartments in CCA tail integrity in response to periodate treatment. Figure S4. Comparison of acid‐phenol and Trizol RNA extraction methods as input into Arabidopsis MSR‐seq libraries. Figure S5. Comparison of RNA samples before (gray) and after (red) treatment with sodium periodate, ribose, sodium tetraborate, and T4 polynucleotide kinase according to the MSR‐seq protocol. Figure S6. 5′ Mapping position of MSR‐seq reads for Arabidopsis organellar tRNAs. Figure S7. Read depth and misincorporation profiles for two tRNA isodecoder families that both show a major 5′ truncation point induced by periodate treatment. Figure S8. 3′ Mapping position of MSR‐seq reads for Arabidopsis organellar tRNAs. Figure S9. Summary of 5′ tRFs. Figure S10. Relationship between nucleotide misincorporation rates in reads with intact CCA tails (aminoacylated tRNAs) vs. reads with CC tails (uncharged tRNAs). Figure S11. Relationship between percentage of reads with intact CCA tails and 5′ truncations as a proxy for “hard‐stop” base modifications. Figure S12. 5′ Mapping position of MSR‐seq reads from periodate‐treated libraries for Arabidopsis nuclear tRNAs. Figure S13. 5′ Mapping position of MSR‐seq reads from periodate‐treated libraries for Arabidopsis organellar tRNAs. Figure S14. Comparison between read abundance for mitochondrial tRNAs (left) and mitochondrial tRNA‐like sequences (right), including t‐elements, tRNA‐Phe‐like sequences, and the orf315 stem‐loop. Table S1. Summary of MSR‐seq libraries generated for this study, including number of reads produced, successfully processed. Table S2. Oligonucleotides used in library construction. [file TPJ-120-2784-s001.zip › tpj17146-sup-0002-Supinfo01.pdf]

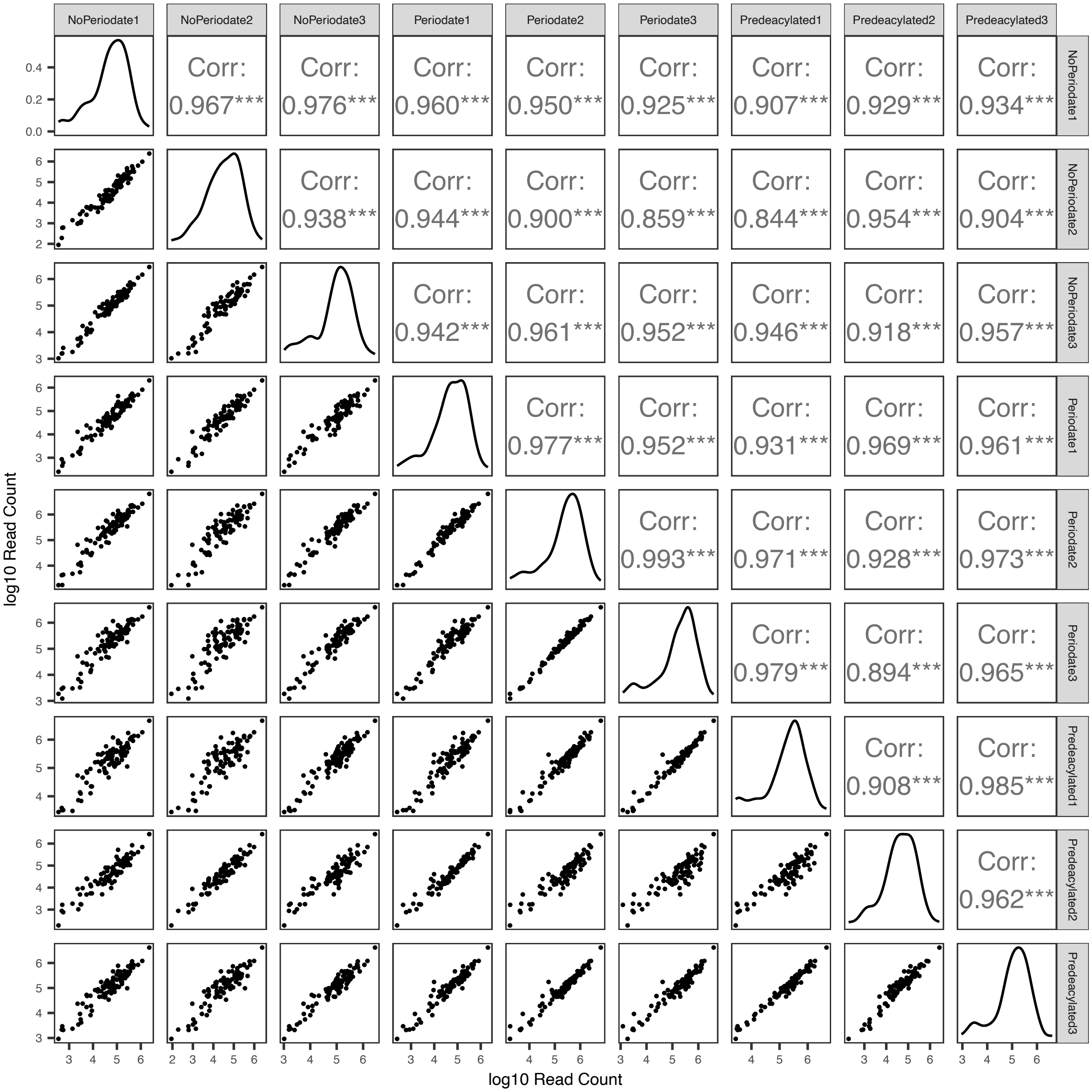

Figure S1. Pairwise matrix showing correlation in read abundance measurements for each library. In scatter plots below the primary diagonal, each point represents the summed read count for all members of an isodecoder family in a given genome (mitochondrial, plastid, or nuclear) on a $\log_{10}$ scale. Reported coefficients above the primary diagonal are from a Pearson correlation analysis on $\log_{10}$ abundance values. The plots on the primary diagonal itself report a read abundance density kernal for each library.

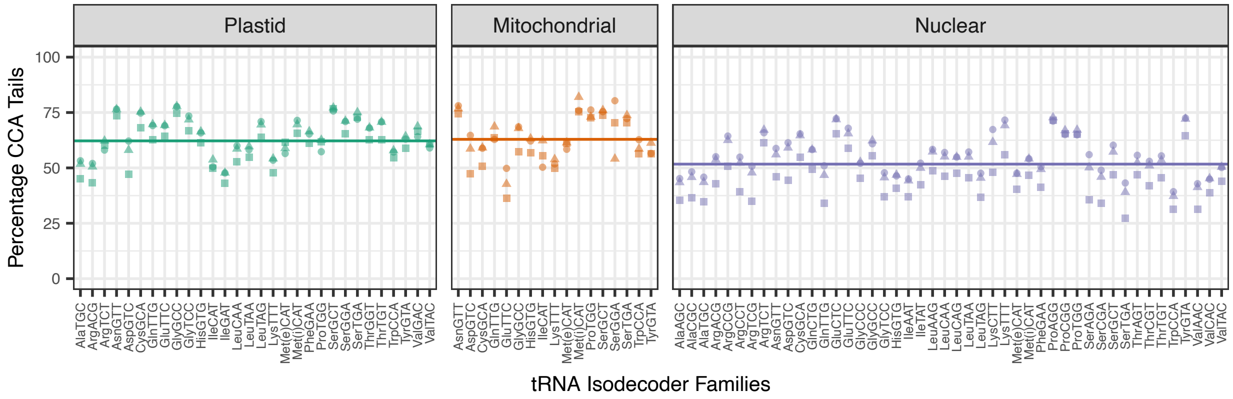

Figure S2. Variation among *Arabidopsis* tRNA isodecoder families and genomic compartments in CCA tail integrity in response to periodate treatment. Reported values represent the percentages of reads with intact CCA tails after excluding reads that lacked more than just a single 3′ nucleotide. Biological replicates are indicated by different shapes. The average frequency of intact CCA tails differs significantly across genomic compartments (p = 2.5e-7; one-way ANOVA), with a lower rate for nuclear-encoded than organellar tRNAs, as indicated by horizontal lines (means) in each panel. Met(e) and Met(i) refer to elongator and initiator tRNA-Met genes, respectively.

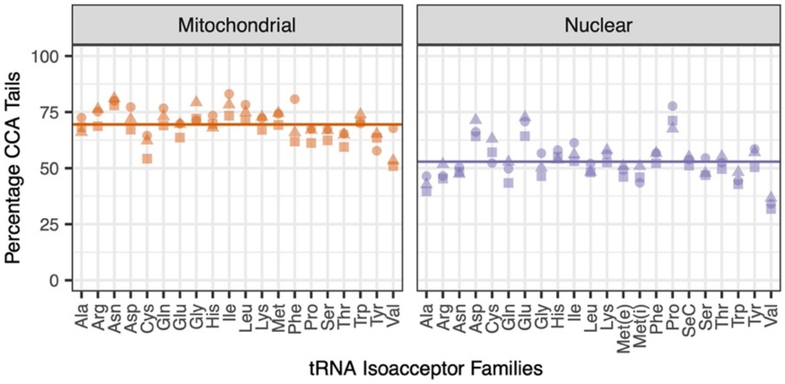

Figure S3. Variation among human cell line (HEK293T) tRNA isoacceptor families and genomic compartments in CCA tail integrity in response to periodate treatment. Data were reanalyzed from a previously published study (Watkins et al. 2022). Reported values represent the percentages of reads with intact CCA tails after excluding reads that lacked more than just a single 3′ nucleotide. Biological replicates are indicated by different shapes. The average frequency of intact CCA tails is significantly higher for mitochondrial tRNAs than nuclear-encoded tRNAs (*p* = 7.8e-9; *t*-test), as indicated by horizontal lines (means) in each panel. Met(e) and Met(i) refer to elongator and initiator tRNA-Met genes, respectively.

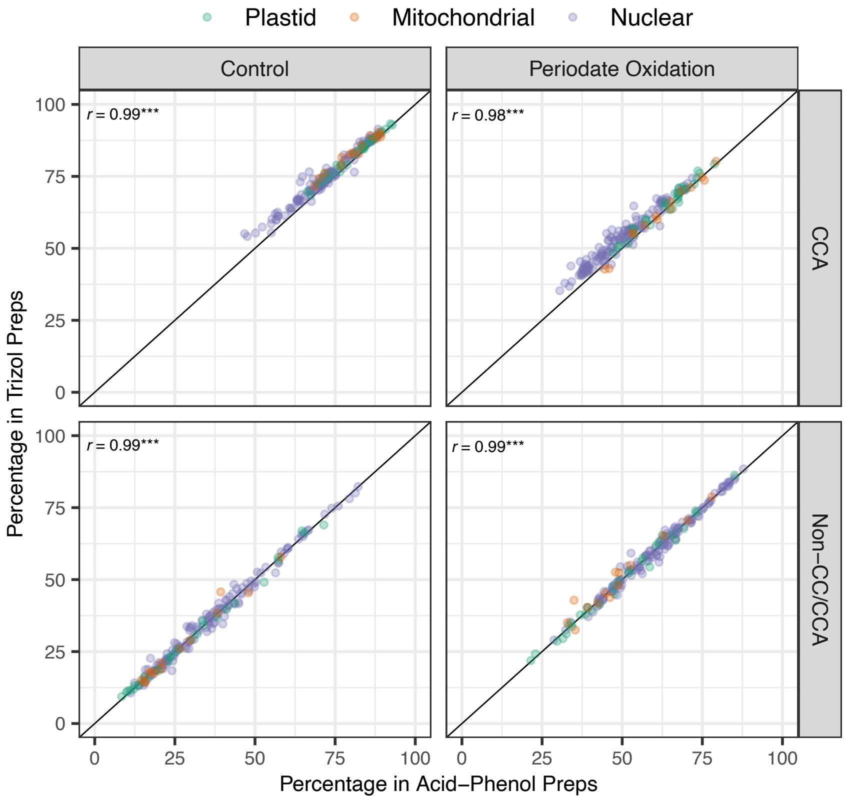

Figure S4. Comparison of acid-phenol and Trizol RNA extraction methods as input into *Arabidopsis* MSR-seq libraries. Reported values for CCA tails (top row) represent the percentages of reads with intact CCA tails after excluding reads that lacked more than just a single 3′ nucleotide. Non-CC/CCA percentages (bottom row) represent the fraction of all tRNA reads that lack two or more nucleotides at their 3′ ends. Each point represents an individual tRNA gene (minimum of 100 reads per gene), with color indicating genome of origin. A one-to-one line is plotted in each panel. Reported *r* values are Pearson correlation coefficients. *** Indicates statistical significance at a $p < 0.001$ threshold.

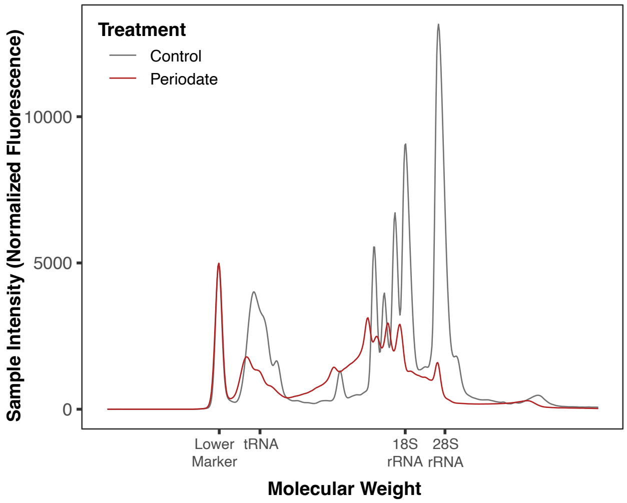

Figure S5. Comparison of RNA samples before (gray) and after (red) treatment with sodium periodate, ribose, sodium tetraborate, and T4 polynucleotide kinase according to the MSR-seq protocol. Data from an Agilent TapeStation 4150 show extensive RNA degradation from periodate treatment.

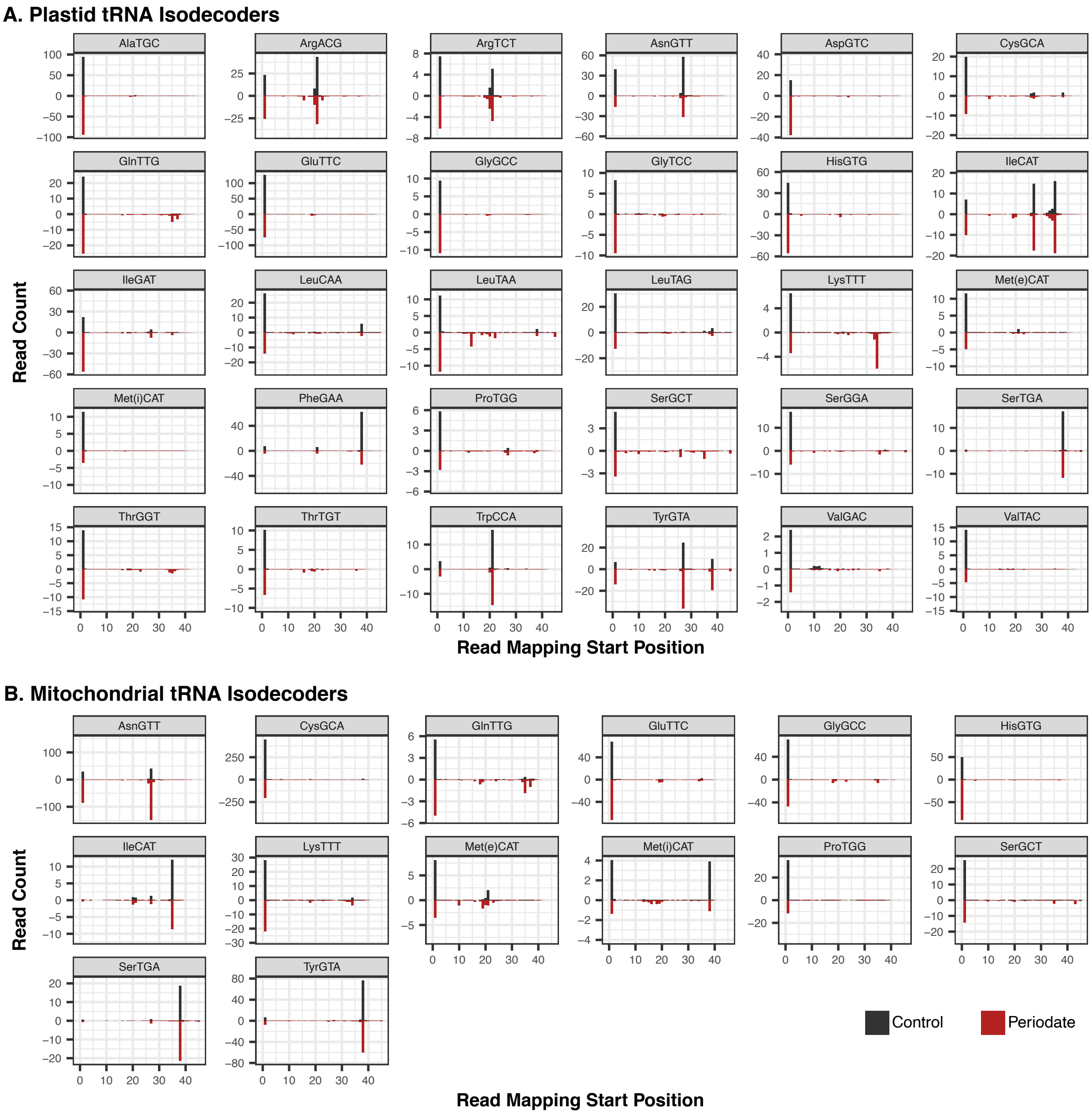

Figure S6. 5′ mapping position of MSR-seq reads for *Arabidopsis* organellar tRNAs. Counts are represented per thousand reads that mapped to the (A) plastid or (B) mitochondrial tRNA gene set (averaged across the three biological replicates). Control libraries (black bars) are shown as positive values above the x-axis, while periodate-treated libraries (red bars) are shown as negative values below the x-axis. Mapping positions are standardized based on the Sprinzl coordinate system (Sprinzl et al. 1998). The mitochondrial AspGTC, SerGGA, and TrpCCA tRNAs are not included in this analysis because a large number of reads from their plastid-derived counterparts ambiguously map to these genes.

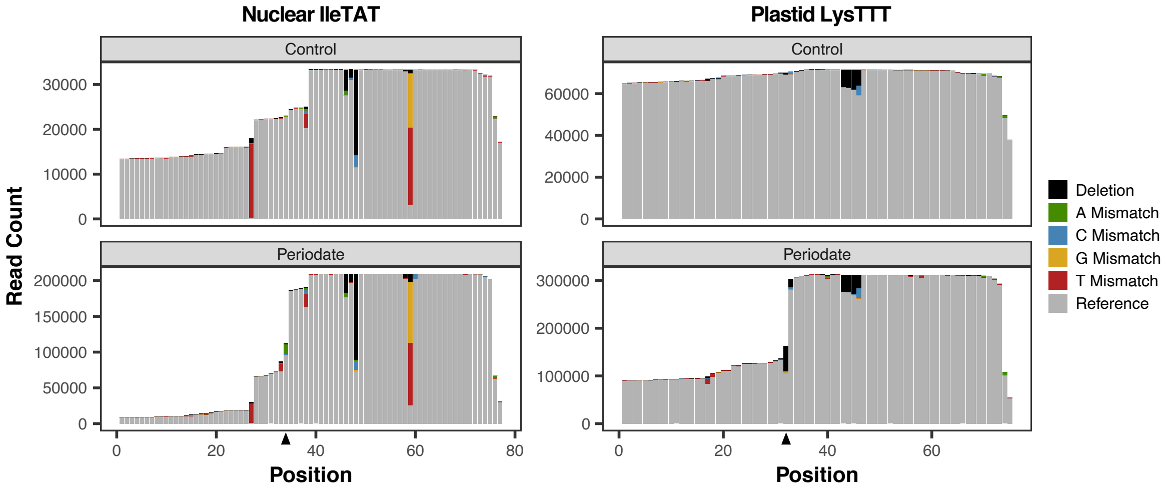

Figure S7. Read depth and misincorporation profiles for two tRNA isodecoder families that both show a major 5′ truncation point induced by periodate treatment. Position 34 in nuclear tRNA-IleTAT (left) and position 32 in plastid tRNA-LysTTT (right) – both of which correspond to position 33 in the Sprinzl coordinate system – show large drops in sequence coverage in periodate-treated samples (bottom) but not in control libraries (top). These positions are indicated by black triangles on the x-axes. In the reads that do cover these positions, there is also a large increase in the frequency of nucleotide misincorporations or deletions. Similar observations in other species (Katanski et al. 2022; Davidsen and Sullivan 2024) have been attributed to effects of periodate on 2-thio-modifications at the adjacent anticodon wobble position (34 in the Sprinzl coordinate system).

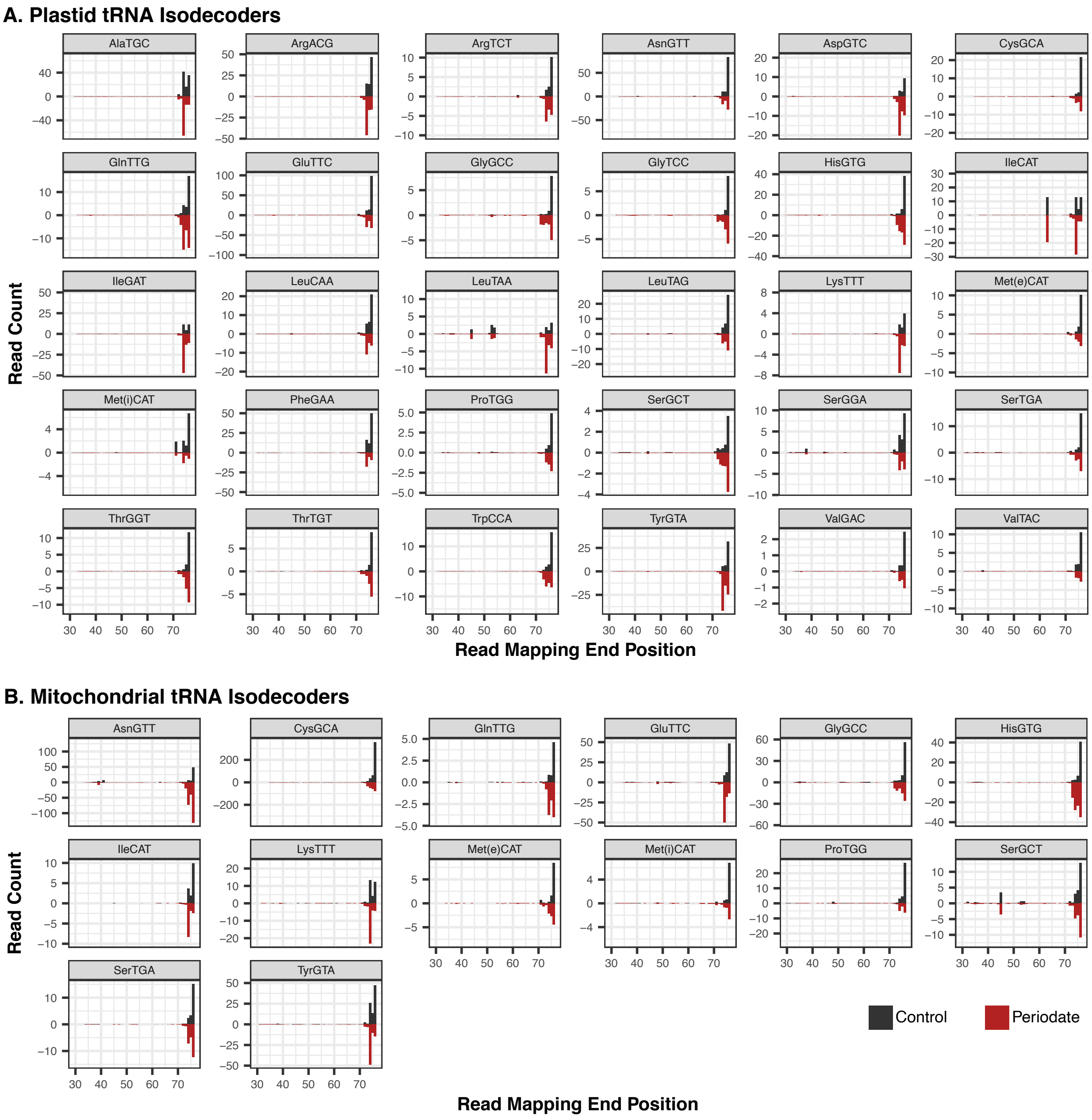

Figure S8. 3′ mapping position of MSR-seq reads for *Arabidopsis* organellar tRNAs. Counts are represented per thousand reads that mapped to the (A) plastid or (B) mitochondrial tRNA gene set (averaged across the three biological replicates). Control libraries (black bars) are shown as positive values above the x-axis, while periodate-treated libraries (red bars) are shown as negative values below the x-axis. Mapping positions are standardized based on the Sprinzl coordinate system (Sprinzl et al. 1998). The mitochondrial AspGTC, SerGGA, and TrpCCA tRNAs are not included in this analysis because a large number of reads from their plastid-derived counterparts ambiguously map to these genes.

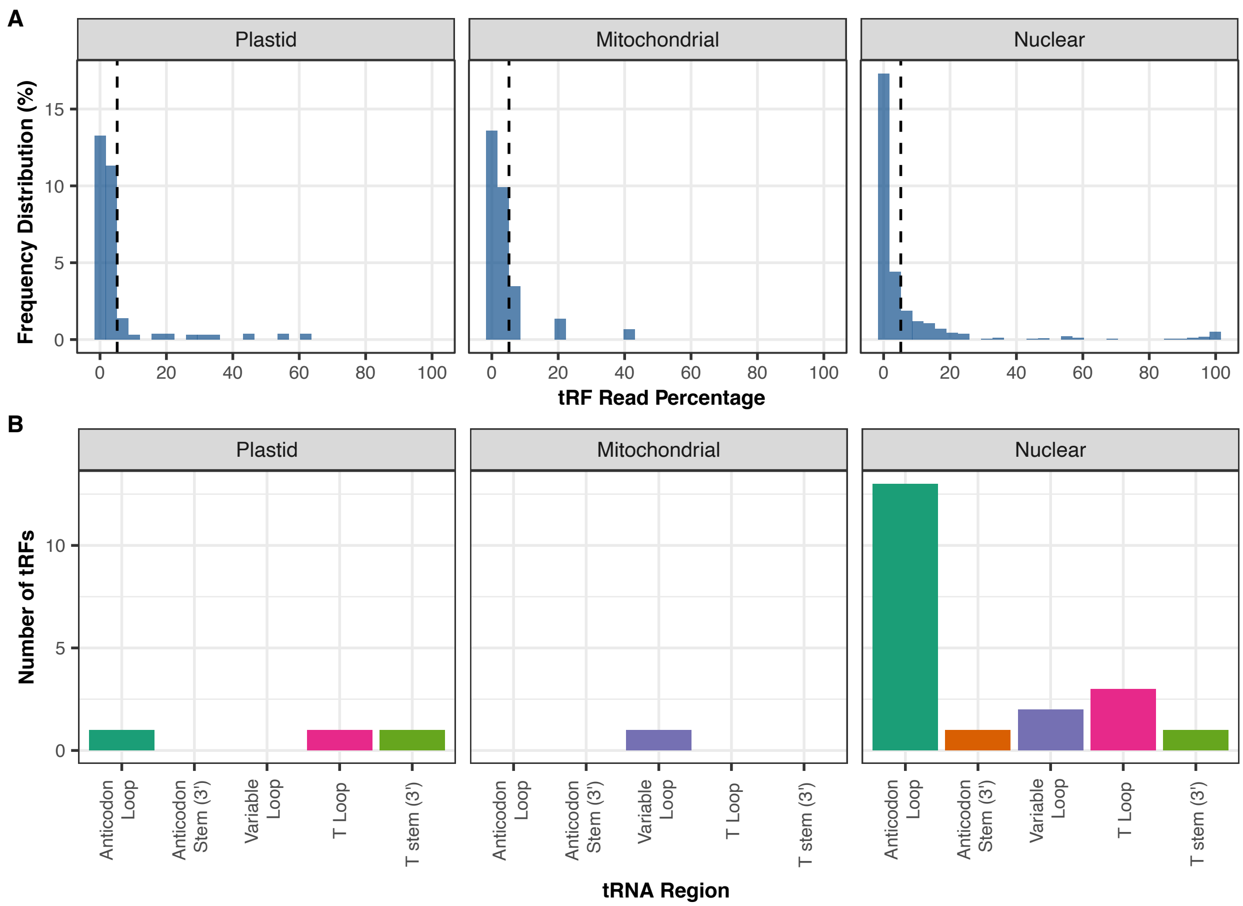

Figure S9. Summary of 5′ tRFs. (A) The proportion of sequence reads represented a minor fraction for most tRNA genes. A threshold of 5% (dashed line) was used to consider fragment a tRF for subsequent analysis. Analysis was limited to genes with a read count > 150. (B) Breakpoints were most often found in the Anticodon loop of nuclear tRNA genes.

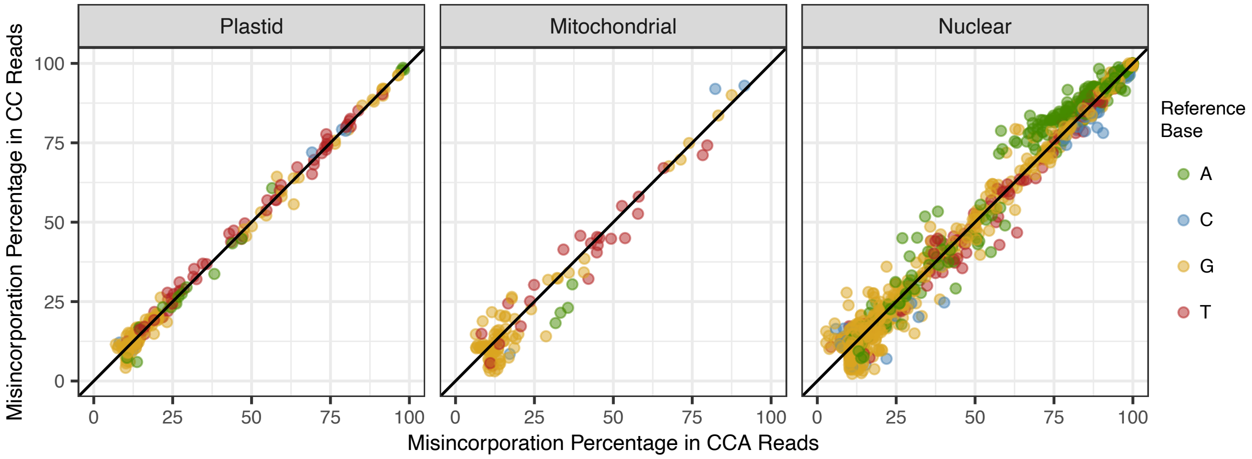

Figure S10. Relationship between nucleotide misincorporation rates in reads with intact CCA tails (aminoacylated tRNAs) vs. reads with CC tails (uncharged tRNAs). Each point represents the combined frequency of nucleotide substitutions and deletions at a specific position in one tRNA gene, averaged across three biological replicates that were treated with periodate. This value is calculated amongst the pool of reads with a full CCA tail (x-axis) or reads that lack their 3′ terminal nucleotide and end in CC (y-axis). Data are only reported for replicates with a read count >100 and for positions with a modification frequency of >10% in at least one sample. Reference tRNA genes are partitioned into panels by genome, and a one-to-one line is plotted in each panel. Misincorporation rates do not differ significantly between CCA and CC reads. As much, they do not provide evidence that aminoacylation levels are dependent on base modifications.

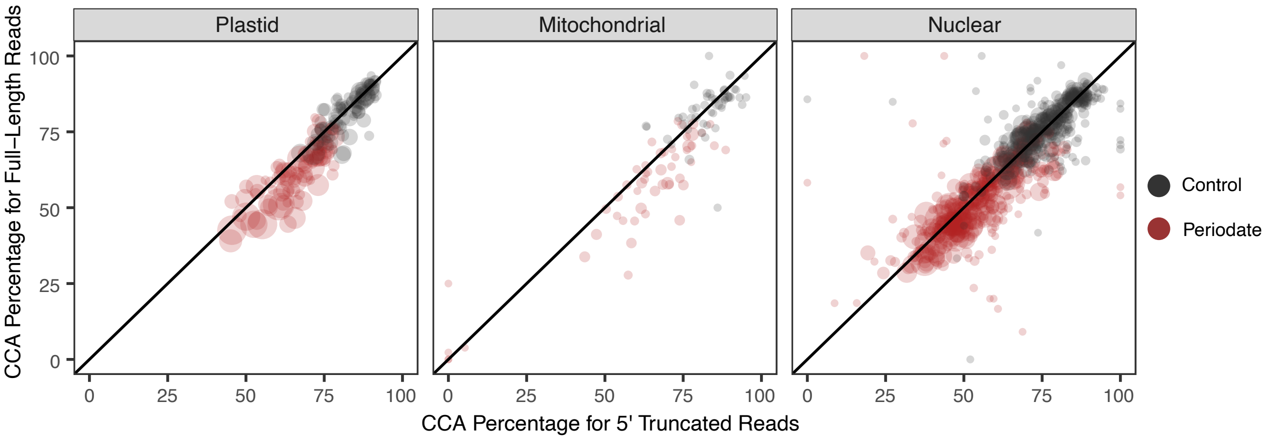

Figure S11. Relationship between percentage of reads with intact CCA tails and 5′ truncations as a proxy for "hard-stop" base modifications. Each point represents the percentage of reads with intact CCA tails after excluding reads that lacked more than just a single 3′ nucleotide. This value is calculated amongst the pool of reads that are truncated at their 5′ end by five or more nt (x-axis) or full-length reads (y-axis). Data are only reported for replicates with a (CC or CCA-containing) read count >150. Reference tRNA genes are partitioned into panels by genome, and a one-to-one line is plotted in each panel. In each of the three genome partitions, CCA percentages are significantly higher for 5′ truncated reads than for full-length reads in periodate-treated libraries (red points) but not in no-periodate controls (gray points). This observation is consistent with hypothesis that the base modifications that result in RT truncations affect tRNA function and increase aminoacylation rates/levels.

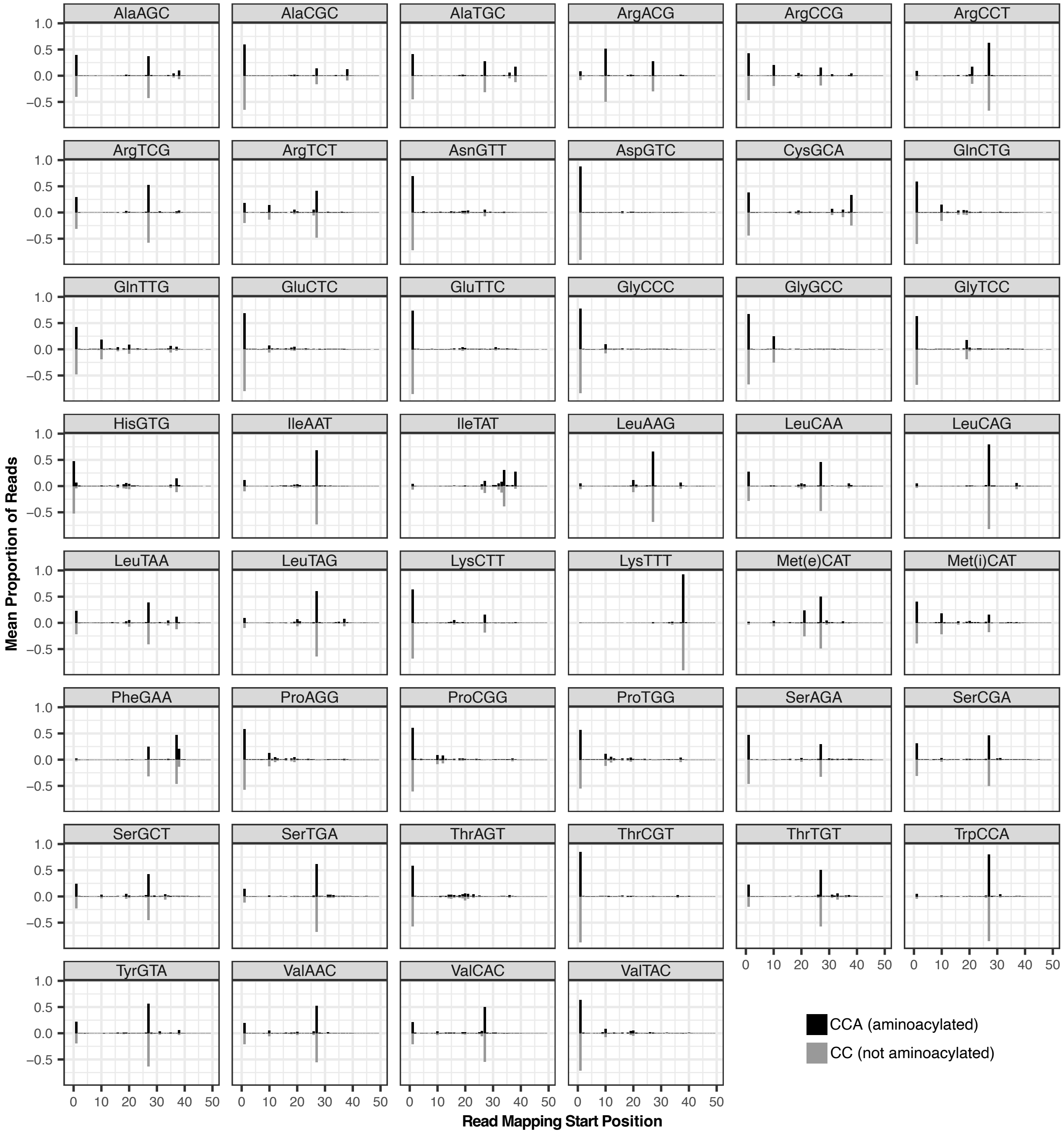

Figure S12. 5′ mapping position of MSR-seq reads from periodate-treated libraries for *Arabidopsis* nuclear tRNAs. Values represent the proportion of reads that mapped to each isodecoder family (averaged across the three biological replicates). Values are calculated separately for reads that retain a full CCA tail (black bars, plotted as positive values above the x-axis) and those that end in CC due to loss of their 3′ terminal nucleotide (gray bars, plotted as negative values below the x-axis). Mapping positions are standardized based on the Sprinzl coordinate system (Sprinzl et al. 1998). The patterns are highly correlated between CCA and CC reads (which should correspond to aminoacylated and uncharged tRNAs, respectively). Cases with small shifts towards a decreased proportion of 5′ truncations in CC reads could suggest a role of base modifications in facilitating aminoacylation (see Figure S11).

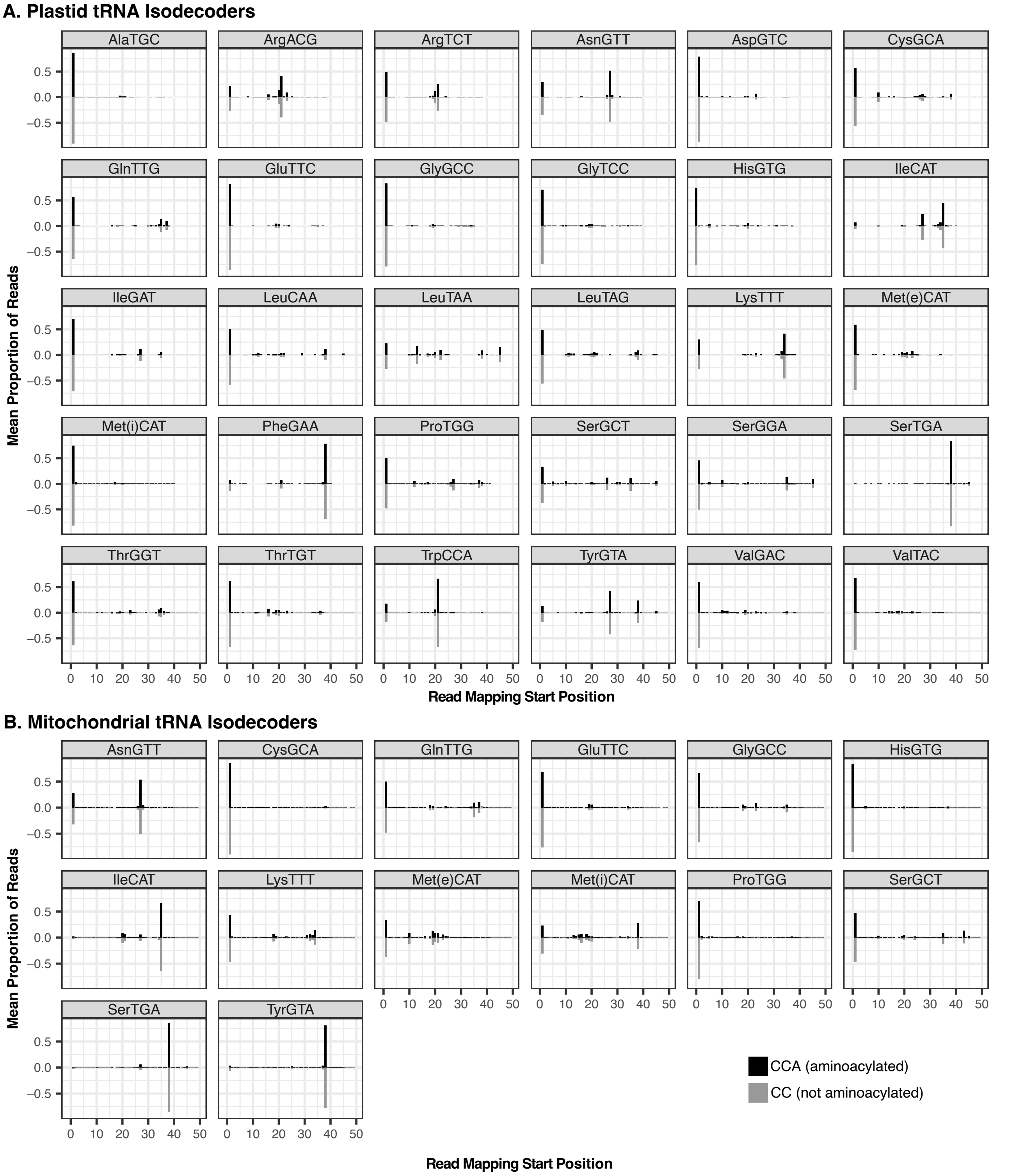

Figure S13. 5′ mapping position of MSR-seq reads from periodate-treated libraries for *Arabidopsis* organellar tRNAs. Values represent the proportion of reads that mapped to the (A) plastid or (B) mitochondrial tRNA gene set (averaged across the three biological replicates). Values are calculated separately for reads that retain a full CCA tail (black bars, plotted as positive values above the x-axis) and those that end in CC due to loss of their 3′ terminal nucleotide (gray bars, plotted as negative values below the x-axis). Mapping positions are standardized based on the Sprinzl coordinate system (Sprinzl et al. 1998). The mitochondrial AspGTC, SerGGA, and TrpCCA tRNAs are not included in this analysis because a large number of reads from their plastid-derived counterparts ambiguously map to these

genes. The patterns are highly correlated between CCA and CC reads (which should correspond to aminoacylated and uncharged tRNAs, respectively). Cases with small shifts towards a decreased proportion of 5′ truncations in CC reads could suggest a role of base modifications in facilitating aminoacylation (see Figure S11).

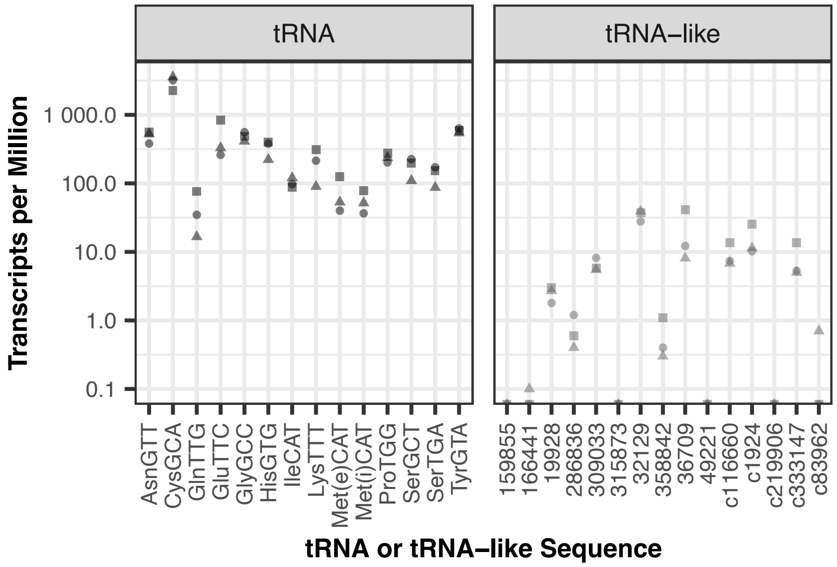

Figure S14. Comparison between read abundance for mitochondrial tRNAs (left) and mitochondrial tRNA-like sequences (right), including t-elements, tRNA-Phe-like sequences, and the *orf315* stem-loop. See Dataset S1 for information on the identity of tRNA-like sequences based on their labeled positions. Three biological replicates from the control (no periodate) samples in experiment 1 are represented by different shapes. Abundances are expressed as transcripts per million mapped to any tRNA in the entire reference database. Three mitochondrial tRNAs (AspGTC, SerGGA, and TrpCCA) were excluded due to substantial levels of ambiguous mapping between mitochondrial and plastid homologs.

Table S1. Summary of MSR-seq libraries generated for this study, including number of reads produced, successfully processed (removal of expected adapter sequencing and merging R1 and R2 reads with BBMerge), and mapped to the tRNA reference set.

NCBI SRA	Experiment	RNA Extraction	Treatment	Rep	Read Pairs	Processed	Mapped
SRR30502882	1	Acid-Phenol	No Periodate	1	17664000	15080940	14110062
SRR30502881	1	Acid-Phenol	No Periodate	2	14443230	12291168	11653350
SRR30502880	1	Acid-Phenol	No Periodate	3	24655596	21714460	20546775
SRR30502892	1	Acid-Phenol	Periodate	1	19364207	14956390	12953944
SRR30502891	1	Acid-Phenol	Periodate	2	66433974	60449437	55430258
SRR30502883	1	Acid-Phenol	Periodate	3	45130569	41270082	37775713
SRR30502879	1	Acid-Phenol	Predeacylated+Periodate	1	49028388	44243871	39008946
SRR30502878	1	Acid-Phenol	Predeacylated+Periodate	2	18369858	14175129	12367300
SRR30502877	1	Acid-Phenol	Predeacylated+Periodate	3	33453924	28715989	25125094
SRR30502887	2	Acid-Phenol	No Periodate (with spike-in)	1	4955018	4670581	4403102
SRR30502886	2	Acid-Phenol	No Periodate (with spike-in)	2	2540908	2379755	2260925
SRR30502876	2	Acid-Phenol	Periodate (with spike-in)	1	5552125	5136589	4642057
SRR30502890	2	Acid-Phenol	Periodate (with spike-in)	2	5809426	5381792	4923051
SRR30502885	2	Trizol	No Periodate (with spike-in)	1	3763956	3589193	3440449
SRR30502884	2	Trizol	No Periodate (with spike-in)	2	4507211	4283316	3970242
SRR30502889	2	Trizol	Periodate (with spike-in)	1	5848749	5507450	5065511
SRR30502888	2	Trizol	Periodate (with spike-in)	2	4461382	4112669	3560700

Table S2. Oligonucleotides used in library construction

Oligo	Sequence	Synthesis (Integrated DNA Technologies)
Capture hairpin oligo (bolded text corresponds to internal MSR barcode)	/5Phos/rACT**GGAA**AGATCGGAAGAGCACACGAT/iBiodT/ AGACGTGTGCTCTTCCGATCT**TTCC**AGrU/3Phos/	250 nmole scale; RNase-free HPLC purification
Second ligation oligo	/5Phos/NNNNNNGATCGTCGGACTGTAGAA/3ddC/	250 nmole scale; HPLC purification
Synthetic spike-in control	rGrGrGrCrCrUrGrUrArGrCrUrCrArGrCrUrGrGrUrUrArGrA rGrCrGrCrArCrGrCrCrUrGrArUrArArGrCrGrUrGrArGrGrU rCrGrGrUrGrGrUrUrCrGrArGrUrCrCrArCrUrCrArGrGrCr CrCrArCrCrA	4 nmole scale; Ultramer RNA oligo (standard desalting)
P5 PCR primer	AATGATACGGCGACCACCGAGATCTACACGTTCAGAGTT CTACAGTCCGACGATC	20 nmole scale, Ultramer DNA oligo (standard desalting)
P7/i7 PCR primer (bolded text corresponds to example i7 index)	CAAGCAGAAGACGGCATACGAGAT**ACGATCAG**GTGACT GGAGTTCAGACGTGTGCTCTTCCGATC*T	4 nmole scale; Ultramer DNA oligo (standard desalting)
