# Supplementary material for: The landscape of Arabidopsis tRNA aminoacylation
Source: Plant J. 2024 Nov 18;120(6):2784–802. doi: 10.1111/tpj.17146 (PMC11658184; doi:10.1111/tpj.17146)
Supplement: Supplementary file 1 — Figure S1. Pairwise matrix showing correlation in read abundance measurements for each library. Figure S2. Variation among Arabidopsis tRNA isodecoder families and genomic compartments in CCA tail integrity in response to periodate treatment. Figure S3. Variation among human cell line (HEK293T) tRNA isoacceptor families and genomic compartments in CCA tail integrity in response to periodate treatment. Figure S4. Comparison of acid‐phenol and Trizol RNA extraction methods as input into Arabidopsis MSR‐seq libraries. Figure S5. Comparison of RNA samples before (gray) and after (red) treatment with sodium periodate, ribose, sodium tetraborate, and T4 polynucleotide kinase according to the MSR‐seq protocol. Figure S6. 5′ Mapping position of MSR‐seq reads for Arabidopsis organellar tRNAs. Figure S7. Read depth and misincorporation profiles for two tRNA isodecoder families that both show a major 5′ truncation point induced by periodate treatment. Figure S8. 3′ Mapping position of MSR‐seq reads for Arabidopsis organellar tRNAs. Figure S9. Summary of 5′ tRFs. Figure S10. Relationship between nucleotide misincorporation rates in reads with intact CCA tails (aminoacylated tRNAs) vs. reads with CC tails (uncharged tRNAs). Figure S11. Relationship between percentage of reads with intact CCA tails and 5′ truncations as a proxy for “hard‐stop” base modifications. Figure S12. 5′ Mapping position of MSR‐seq reads from periodate‐treated libraries for Arabidopsis nuclear tRNAs. Figure S13. 5′ Mapping position of MSR‐seq reads from periodate‐treated libraries for Arabidopsis organellar tRNAs. Figure S14. Comparison between read abundance for mitochondrial tRNAs (left) and mitochondrial tRNA‐like sequences (right), including t‐elements, tRNA‐Phe‐like sequences, and the orf315 stem‐loop. Table S1. Summary of MSR‐seq libraries generated for this study, including number of reads produced, successfully processed. Table S2. Oligonucleotides used in library construction. [file TPJ-120-2784-s001.zip › tpj17146-sup-0003-Supinfo02.pdf]

MSR-seq Protocol (ver. 6/17/24)

Protocol for generating MSR-seq libraries to capture tRNA sequences and assess tRNA-specific aminoacylation rates. Based on the paper by Watkins et al. 2022: https://www.nature.com/articles/s41467-022-30261-3

Reagents

Enzymes

- T4 RNA Ligase I. New England Biolabs M0204L
  - Includes 10x buffer, 50% PEG 8000 and 10 mM ATP stock
- RNase H. New England Biolabs M0297L
- CIAP (Calf Intestinal Alkaline Phosphatase). 20 U/ul. Invitrogen 18009019
- SuperScript IV VILO Master Mix. Invitrogen 11756050
- T4 Polynucleotide Kinase (PNK). New England Biolabs M0201S
- Q5 High-Fidelity DNA Polymerase. New England Biolabs M0491S

Chemicals/Solutions

- Nuclease-Free Water. Invitrogen AM9937
- Tris-HCl pH 9.0 (1M), RNase-free. Boston Bioproducts BBT90500ML
- Tris-HCl pH 7.4 (1M), RNase-free. Boston Bioproducts BBT74500ML
- Sodium acetate (NaOAc), pH 5.2 (3M), RNase-free. Thermo-Scientific Chemicals J61928AE
- Sodium acetate (NaOAc), pH 4.5 (1M), RNase-free. Thermo-Scientific Chemicals J63669AE
- EDTA pH 8.0 (0.5 M). Invitrogen AM9262
- Magnesium chloride (NaCl) 1M, RNase-free. Invitrogen AM9530G
- Sodium chloride (NaCl) 5M, RNase-free. Invitrogen AM9760G
- dNTPs 100 mM. Thermo-Fisher. FERR0182
- Acid-Phenol:Chloroform, pH 4.5 (with 1 IAA, 125:24:1). Invitrogen AM9720
- Dimethyl Sulfoxide (DMSO). Millipore-Sigma 317275100ML
- Ethanol, 200 Proof. Pharmco 111000200
- Sodium dodecyl sulfate (SDS), 10% stock
- Tween 20. VWR 0777-1L
- Hexaamminecobalt(III) chloride, 99%, Thermo-Scientific Chemicals 405310050
  - Prepared 50 mM stock in RNase-free dH2O, stored at -20 C
- D(-)-Ribose, 99+%. Thermo-Scientific Chemicals 132361000
  - Prepared 1M and 0.6M stocks in RNase-free dH2O, stored at -20 C
- Sodium periodate, 99.8+%. Thermo-Scientific Chemicals 419611000
- Sodium tetraborate decahydrate. Sigma-Aldrich S9640

Other

- Dynabeads MyOne Streptavidin C1. Invitrogen 65001
- SPRI beads for DNA clean-up (lab homebrew protocol)
- 96-well magnetic rack
- BluePippin 3% Agarose Gel Cassette with Marker Q3
- Qubit dsDNA and RNA Assay kits
- TapeStation RNA and D1000 DNA reagents
- Liquid N_2

Plasticware

- DNA LoBind Tubes 1.5 ml. Eppendorf 022431021

- Snapstrip PCR Tubes with Dome Cap. 0.2 ml. BioExpress (GeneMate) T-3035-1
- Micropestles

Oligonucleotides

- Capture Hairpin Oligo (CHO)
  - /5Phos/rACT**GGAA**AGATCGGAAGAGCACACGAT/iBiodT/AGACGTGTGCTCTTCCGATCT**TTCC**AGrU/3Phos/
  - Bolded sequence (GGAA/TTCC) is an internal barcode for multiplexing. Multiple can be ordered.
  - 250 nmole RNA oligo (most bases are DNA, but RNA synthesis because of the terminal ribonucleotides) (IDT)
  - RNase-free HPLC purification
  - Modifications: ribonucleotides, phosphates, and biotin
  - Dissolve in RNase-free dH2O at 50 uM concentration

- Lig2_MSR-seq
  - /5Phos/NNNNNNGATCGTCGGACTGTAGAA/3ddC/
  - 250 nmole DNA oligo (IDT)
  - HPLC purification
  - Modifications: 5’ phosphate and 3’ dideoxy terminator
  - Dissolve in RNase-free dH2O at 50 uM concentration

- MSR_P5amp (PCR primer)
  - AATGATACGGCGACCACCGAGATCTACACGTTCAGAGTTCTACAGTCCGACGATC
  - 20 nmole Ultramer DNA Oligo (IDT)
  - Standard desalting purification
  - Dissolve in dH2O at 10 uM concentration (master stock can be kept at 100 uM)

- p7/i7 (PCR primer)
  - CAAGCAGAAGACGGCATACGAGAT**ACGATCAG**GTGACTGGAGTTCAGACGTGTGCTCTTCCGATC*T
  - Bolded sequence is an i7 barcode for multiplexing. Multiple can be ordered.
  - 4 nmole Ultramer DNA Oligo (IDT)
  - Standard desalting purification
  - Modification: 3’ phosphorothioate bond
  - Dissolve in dH2O at 10 uM concentration (master stocks can be kept at 100 uM)

- Synthetic tRNA spike-in control
  - rGrGrGrCrCrUrGrUrArGrCrUrCrArGrCrUrGrGrUrUrArGrArGrCrGrCrArCrGrCrCrUrGrArUrArArGrCrGrUrGrArGrGrUrCr GrGrUrGrGrUrUrCrGrArGrUrCrCrArCrUrCrArGrGrCrCrCrArCrCrA
  - Based on Bacillus subtilis trnI (NC_000964.3:31932-32008). Chosen because it is not expected to have similarity to the plant tRNAs we have been working with.
  - Purpose is to use as a control to see if complete elimination of 3’ nucleotide occurs for naked tRNA upon periodate treatment and whether full-length tRNAs are recovered in the absence of periodate treatment.
  - 4 nmole Ultramer RNA Oligo (IDT)
  - Standard desalting purification
  - Dissolve in RNase-free dH2O at 10 uM concentration (~250 ng/ul). Master stock can be kept at 100 uM.
  -

Buffers

Lysis Buffer

- 300 mM NaOAc pH 4.5

- 10 mM EDTA, pH 8.0
- 1% SDS
- In RNase-free dH2O

High-Salt Wash Buffer (note that the original publication does not mention Tween, but Chris Katanski confirmed that it should be included, pers. comm.)

- 1M NaCl
- 20 mM Tris-HCl pH 7.4
- 0.1% Tween 20
- In RNase-free dH2O

Low-Salt Wash Buffer

- 0.1M NaCl
- 20 mM Tris-HCl pH 7.4
- In RNase-free dH2O

RNA Extraction

This acid-phenol extraction is performed under low pH to prevent deacylation of tRNAs so that MSR-seq can subsequently be used to assess native aminoacylation state. The protocol is largely based on Zaborske et al. 2009 JBC (https://www.jbc.org/article/S0021-9258(20)30613-X/fulltext) with inclusion of 1% SDS to aid in plant cell lysis (Laurence Drouard pers. comm.).

- ~20 mg of fresh Arabidopsis leaf tissue ground under liquid N_2 in Eppendorf LoBind tube
- Add 500 ul Lysis Buffer
- Use pipet tip and vortexed to resuspend ground tissue.
- Add 500 ul of acid-phenol/chloroform/isoamyl-alcohol (125:24:1) pH 4.5.
- Vortex 3 times for ~30 sec each, placing on ice in between.
- Centrifuge: 15 min, 20k rcf, 4 C.
- Recover as much of aqueous phase as possible without pulling from organic phase.
- Add another 500 ul of acid-PCI pH 4.5 (see above).
- Mix by vortexing.
- Once again, centrifuge: 15 min, 20k rcf, 4 C.
- Remove 400 ul of aqueous phase, and add to 1080 ul (2.7x volumes) ethanol.
- Mix by vortexing.
- Store at -20 C for 30 min to precipitate RNA.
- Centrifuge: 10 min, 18.5k rcf, 4 C.

- Remove supernatant and dissolve pellet (no need to dry) in 200 ul of the following buffer.
  - 300 mM NaOAc pH 4.5
  - 10 mM EDTA, pH 8.0
  - In RNase-free dH2O

- Add 540 ul (2.7x volumes) molecular-grade EtOH. Mix by vortexing.

- Store at -20 C for 30 min to precipitate RNA.

- Centrifuge: 10 min, 18.5k rcf, 4 C.

- Remove supernatant and briefly air-dry pellet.

- Dissolve pellet in 20 ul of 10 mM NaOAc pH 4.5.

- Check RNA quantity and integrity with Qubit and TapeStation. RNA should show minimal evidence of degradation based on substantially stronger (ideally 2x) LSU (28S) rRNA peak compared to SSU (18S) rRNA peak. Note that in the profile below (acid-phenol extraction of Arabidopsis Col-0 rosette RNA), the migration is a little off, so sizing estimates may be inaccurate, but the profile should generally look like this.

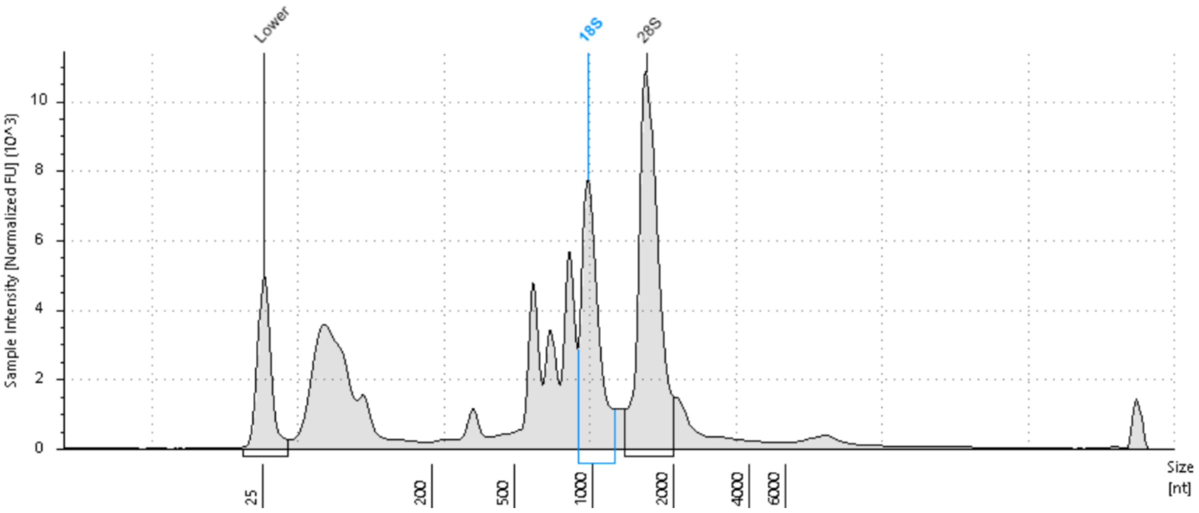

Deacylation

This step is not necessary for library prep when the periodate treatment (see below) is performed because the basic conditions of the sodium tetraborate incubation should remove amino acids from charged tRNAs. But it must be performed as part of certain control reactions (described in “Periodate Treatment” section below) or if MSR-seq is being done without trying to assay aminoacylation status.

- Dilute 2 ug of the above RNA sample to a volume of 50 ul in a final concentration of 50 mM Tris-HCl pH 9.0 in 0.2 ml PCR tube. (Note: This quantity of RNA should be sufficient for at least two downstream libraries: e.g., with and without periodate treatment)

- If including a spike-in synthetic tRNA control, add it at this stage as part of the 50 ul volume. The 10 uM stock can first be diluted 250-fold in 10 mM NaOAc pH 4.5. Adding 1 ul of this

diluted stock will correspond to ~1 ng of the internal control, which has served as an appropriate amount in our runs.

- Incubate at 37 for 30 min.

- Ethanol precipitation
    - First add 10 ul 1M NaOAc pH 4.5 to neutralize the deacylation reaction.
    - Then add 3 ul of 3 M NaOAc pH 5.2 to bring salt concentration up to ~0.3M.
    - Add 170 ul ethanol and mix by vortexing.
    - Incubate at -20 C for 30 min.
    - Centrifuge: 10 min, 18.5k rcf, 4 C
    - Aspirate supernatant and wash in 500 ul 80% EtOH
    - Centrifuge: 5 min, 18.5k rcf, 4 C
    - Aspirate supernatant and very briefly air-dry.
    - Dissolve in 15 ul RNase-free dH2O.

- Quantify with Qubit RNA assay

Periodate Treatment

The goal of this step is to remove the 3' nucleotide of any tRNAs that are not aminoacylated (the amino acid protects the 3' end of charged tRNAs). It proceeds directly from extracted RNAs for the standard MSR-seq library protocol (i.e., no deacylation). Control libraries can also be run by skipping this step and working with a deacylated sample (no 3' nucleotide removal expected) or by deacylating the sample prior to performing this step (complete 3' nucleotide removal expected). See "Deacylation" step above. Note: this treatment introduces significant degradation of RNA based on a pre/post test that I ran.

- Start with 500 ng of RNA sample in 8 ul volume of 10 mM NaOAc pH 4.5 in 0.2 ml PCR tube.

- If including a spike-in synthetic tRNA control, add it at this stage as part of the 8 ul volume. The 10 uM stock can first be diluted 250-fold in 10 mM NaOAc pH 4.5. Adding 1 ul of this diluted stock will correspond to ~1 ng of the internal control, which has served as an appropriate amount in our runs.

- Add 1 ul of freshly prepared 150 mM sodium periodate in RNase-free dH2O (128 mg in 4 ml; this is a much larger volume than needed but it accommodates weighing the small amount of the dry chemical). Mix by vortexing. Sodium periodate is very hazardous. Take appropriate precautions in handling and disposal.

- Incubate at room temp for 30 min.

- Add 1 ul of 0.6M ribose. Mix by vortexing.

- Incubate at room temp for 5 min.

- Add 5 ul of freshly prepared 100 mM sodium tetraborate decahydrate in RNase-free dH2O (153 mg in 4 ml; this is a much larger volume than needed but it accommodates weighing the small amount of the dry chemical). Mix by vortexing. (Note: The paper states that this should be freshly prepared, but Chris Katanski said that fresh preparation is not necessary for sodium tetraborate [only necessary for periodate solution above], but I prepared it fresh anyway.)

- Incubate at 45 C for 30 min.

- Add 5 ul of the following master mix and mix by pipetting.
    - 200 mM Tris-HCl pH 7
    - 40 mM MgCl2
    - 4 U/ul T4 PNK
    - In RNase-free dH2O

- Incubate at 37 C for 20 min followed by 65 C for 10 min.

Capture Hairpin Oligo (CHO) Ligation

This step ligates the CHO to the 3' end of the RNAs.

- Prepare enough of the following master mix to have 29 ul per reaction (sample), including enough excess for pipetting error. Add reagents in the order specified. Mix well by pipetting (PEG makes for highly viscous solution).

**Reagent**	**Volume (ul)**
10x RNA Ligase I Buffer	5
50% PEG 8000	15
5 mM ATP (diluted two-fold from 10 mM stock in RNase-free H2O)	0.5
hexaammine cobalt (III) chloride (50 mM Stock)	1
DMSO	2.5
RNA Ligase I	5
**Master Mix per Reaction**	**29**

- Add 20 ul of RNA or RNase-free dH2O depending on sample type to 29 ul of the above master mix in 0.2 PCR tube.
    - The 20 ul output of the above periodate treatment (500 ng RNA) can be added directly.
    - If using a deacylated RNA sample that was not periodate-treated, add RNA in 20 ul of RNase-free dH2O. I used 1 ug (instead of 500 ng) for these samples, reasoning that they were more intact and, thus, had fewer small pieces for ligation).
    - Add 20 ul of RNase-free dH2O if performing a no-template control.

- Add 1 ul of 50 uM CHO.

- Mix by pipetting. (In my run, I used the IKA vortexer for ~10 s at 2000 rpm. Those preps largely worked, but I am worried the vortex is not strong enough to distribute the CHO through the viscous sample).

- Incubate at 16 C overnight.

Streptavidin Bead Binding

This step binds the biotin-labeled CHO (and ligated RNAs) to streptavidin-coated magnetic beads. This will facilitate repeated purification of products after downstream enzymatic and chemical steps.

- Add 50 ul RNase-free dH2O to reduce viscosity.

- Add 20 ul of MyOne C1 Dynabeads (Note: the protocol says 12 ul, but I wanted a bit more bead volume to work with in downstream purifications. It would make sense to reduce this volume to a lower amount per sample if multiple ligated samples with different CHO barcodes were being pooled at this stage for multiplexing).

- Mix on IKA vortexer.

- Incubate at room temp for 15 min.

- Wash in PCR tubes with High-Salt and then Low-Salt Buffers. For each wash, pellet beads with magnetic rack, remove supernatant, add 190 ul wash buffer. Remove from magnet and resuspend by pipetting. Remove final supernatant. Wash removals and resuspensions were done with multichannel pipets. New reagents were added with individual pipets. This is the standard bead wash protocol for later protocol steps.

Dephosphorylation

This step removes the 3' phosphate from CHO, which was present to prevent self-ligation in previous ligation step but must be absent for reverse transcription to extend 3' end.

- Prepare a master mix with the following components for each reaction (including enough excess for pipetting error).

**Reagent**	**Volume (ul)**
10x CIAP reaction buffer	5
CIAP (10x dilution in provided dilution buffer)	1
RNase-free dH2O	44
**Master Mix per Reaction**	**50**

- Add 50 ul of the above master mix to each bead pellet and resuspend by pipetting.

- Incubate at 37 C for 30 min.

Reverse Transcription

This step extends the CHO to generate cDNA from the ligated RNA molecule.

- Perform High-Salt Buffer and then Low-Salt Buffer washes on dephosphorylation reactions as above.

- Resuspend beads in 20 ul RNase-free dH2O by pipetting. Note: I was concerned that beads were starting to dry out as I got to later steps. Consider approaches that remove final wash just before resuspension.

- Add 5 ul SuperScript IV VILO 5x master mix, and mix on IKA vortexer.

- Incubate for 10 min at 55 C followed by 12 hr at 35 C.

RNase H Digestion

This step degrades the original RNA template that is now hybridized with the cDNA produced by reverse transcription above.

- Perform High-Salt Buffer and then Low-Salt Buffer washes on reverse transcription reactions as above.

- Prepare a master mix with the following components for each reaction (including enough excess for pipetting error).

Reagent	Volume (ul)
RNase H	4
10x Buffer	5
RNase-Free dH2O	41
**Master Mix per Reaction**	**50**

- Add 50 ul of the above master mix to resuspend each pellet. Mix on IKA vortexer.

- Incubate at 37 C for 15 min.

Periodate Treatment

This step modifies the 3' ribonucleotide on unligated CHOs such that they can't ligate to the second ligation adapter in next step.

- Perform High-Salt Buffer and then Low-Salt Buffer washes on RNase H reactions as above.

- Resuspend in 40 ul RNase-free dH2O.

- Freshly prepare stock of 250 mM sodium periodate, 0.5M NaOAc pH 5.2. To do this, first dissolve the sodium periodate in RNase-free dH2O (160 mg in 2.5 ml; this is a much larger volume than needed but it accommodates weighing the small amount of the dry chemical) and then add the appropriate amount of 3M NaOAc (500 ul) to reach 0.5M final concentration. If NaOAc is added first, there is very rapid precipitation out of solution. Even when periodate is added first, precipitation will occur, so work quickly after stock is prepared. Sodium periodate is very hazardous. Take appropriate precautions in handling and disposal.

- Add 10 ul of the above stock to each sample and mix with IKA vortexer.

- Incubate at room temp for 30 min.

- Add 10 ul of 1M ribose stock and mix with IKA vortexer.

- Incubate at room temp for 5 min.

Second Adapter Ligation

This step adds the Lig2_MSR-seq adapter to the 3' end of the cDNA generated from reverse transcription of the CHO-ligated RNAs.

- Perform High-Salt Buffer and then Low-Salt Buffer washes on periodate/ribose treated samples as above.

- Prepare a master mix with the following components for each reaction (including enough excess for pipetting error).

**Reagent**	**Volume (ul)**
RNase-Free dH2O	2.75
10x RNA Ligase I Buffer	5
50% PEG 8000	25
5 mM ATP (diluted two-fold from 10 mM stock in RNase-free H2O)	0.5
hexaammine cobalt (III) chloride (50 mM Stock)	1
DMSO	3.75
RNA Ligase I	10
**Master Mix per Reaction**	**48**

- Add 48 ul of the above master mix to each tube.

- Then add 2 ul of the 50 uM second ligation oligo (Lig2_MSR-seq).

- Notes and thoughts about addition and mixing of second ligation reagents: I pulled off the final wash with a multichannel, and then added the master mix individually to each tube. This means the later tubes had more time waiting with naked pellets. After adding the master mix to each tube, I tried vortexing (on IKA vortexer) the PCR strip tubes. But this didn't seem have an effect in dispersing the bead pellet through the viscous solution. So tube-by-tube, I added the 2 ul of oligo and, before going on to the next tube, I resuspended the beads and mixed in the oligo by pipetting. This required physically scraping beads off the tube wall with the pipet tip. After doing this for all tubes, I did another IKA vortex (not sure that was important). The resuspending process was quite slow. This raises the question as to whether it's problematic that the beads are spending a long time in the master mix without the oligo. Conversely, if the oligo gets added without resuspending the beads ahead of time or quickly thereafter, that might be a concern. In the future. It might be best to best to process tube-by-tube, removing the wash, adding the master mix, adding the oligo, and then mixing before going on to the next tube.

- Incubate at room temp overnight.

- Dilute each sample with 50 ul RNase-free dH2O to reduce viscosity.

- Perform High-Salt Buffer and then Low-Salt Buffer washes as above.

- Remove low-salt wash buffer from tubes one-by-one followed immediately by resuspension in 15 ul of RNase-free dH2O. Note the protocol says only 6 ul per library in the pool. However, because I only had one library in each sample and did not pool, I wanted a larger volume to work with.

PCR Library Amplification

This step amplifies the library molecules and incorporates Illumina adapters, including i7 index for multiplexing.

- Freeze beads at -20 C overnight. I have seen multiple cases where PCR amplification has performed better after the beads have gone through a freeze-thaw cycle, yielding a larger fraction of library molecules in the tRNA size range (as opposed to the large adapter peak). The reason for this improved amplification after freezing is not clear, but it appears to be a repeatable pattern.

- Prepare a master mix with the following components for each reaction (including enough excess for pipetting error). In addition to MSR-seq samples prepared above, it is advisable to perform a negative control (dH2O template) and a positive control (dilute template from a previously amplified library with compatible adapters).

Reagent	Volume (ul)	Final Conc
5X Q5 Buffer	10	1x
100 mM dNTPs	0.1	200 µM
10 µM MSR-P5_Amp Primer	2.5	0.5 µM
Q5 High-Fidelity DNA Polymerase	0.5	0.02 U/µl
dH2O	31.4	
**Master Mix per Reaction**	44.5	

- Add 44.5 ul of master mix to a 0.2 ml PCR tube for each reaction that will be performed.

- Add 2.5 ul of 10 uM IDT i7 primer (different i7 for each reaction for subsequent multiplexing)

- Add 3 ul of MSR-seq bead slurry or other control template to each reaction.

- Thermal cycling
  - 3 min at 98 C
  - 15 cycles of: 10 s at 98 C, 15 s at 55 C, 20 sec 72 C
  - 5 min at 72 C
  - Hold at 4 C

  - Note that 15 cycles is the high end of the range suggested in the protocol. It is possible that this could be reduced, but my yields were not very high even with this cycle number.

- Perform clean-up with standard SPRI bead protocol (1.6x bead ratio), eluting each sample in 20 ul 10 mM Tris-HCl pH 7.4. Note that samples with the streptavidin beads (and SPRI beads) pelleted differently in final elution than the control PCR products with only SPRI

beads. This may lead to a small amount of bead carryover in the elution volume for the main samples with streptavidin beads. But this small amount of remaining beads should get removed in a later clean-up after pooling.

- Check library quantity and size distribution with Qubit dsDNA and TapeStation D1000 assays. Libraries should look something like the following. The large peaks is an adapter artefact that is also produced in control libraries with no reverse transcriptase or no original RNA sample. The two secondary peaks like correspond to full-length tRNAs (larger fragments) and common truncations of tRNAs at hard-stop modifications (smaller fragments).

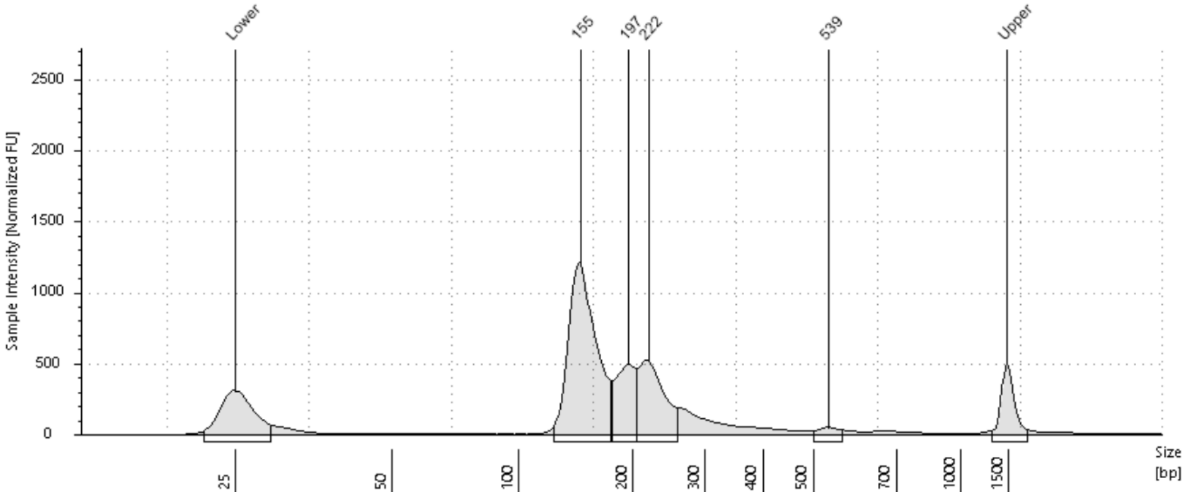

BluePippin Library Size Selection

This step removes adapter artefacts to sequence inserts within the target size range.

- Pool libraries into a single sample. They already contain i7 barcodes for multiplexing, and doing size selection on a single pool will avoid differential representation based on lane-to-lane variation in size selection boundaries.

- Reduce total volume to 30 ul by performing a (1.6x) SPRI bead clean-up, eluting in 30 ul 10 mM Tris-HCl pH 7.4.

- Run BluePippin 3% cassette with Q3 Marker following standard protocol and setting size selection to “Range” with 170-275 bp boundaries.

- Perform (1.6x) SPRI bead clean-up on recovered size-selected product, eluting in the desired quantity of 10 mM Tris-HCl pH 7.4 based on expected yield and preferred library concentration and volume.

- Check library quantity and size distribution with Qubit dsDNA and TapeStation D1000 assays. The library should no longer contain noticeable adapter artefacts.

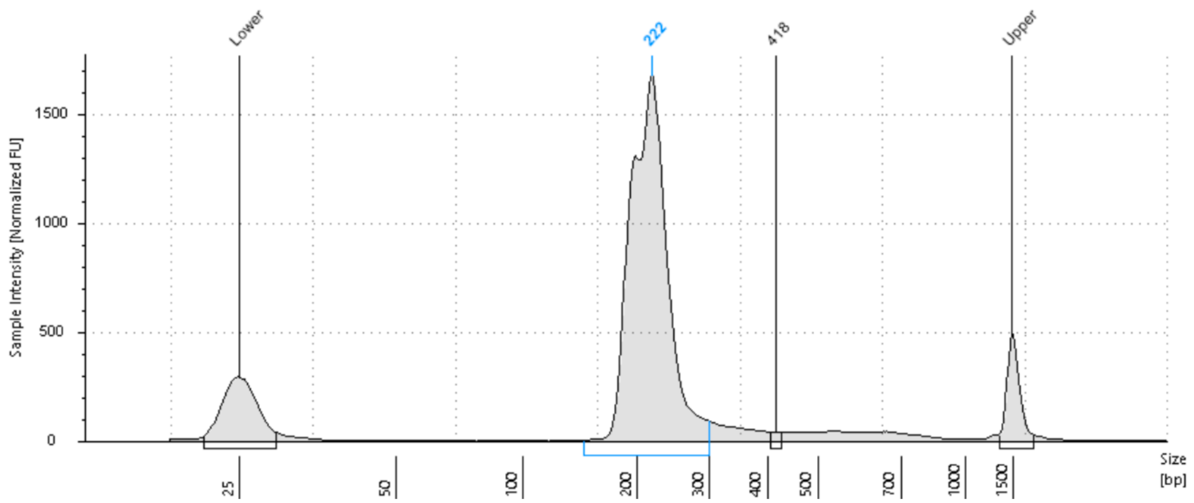

Lower
222
418
Upper
Sample Intensity [Normalized FU]
1500
1000
500
0
25
50
100
200
300
400
500
700
1000
1500
Size [bp]
